# Supplementary material for: Sputnik-V reactogenicity and immunogenicity in the blood and mucosa: a prospective cohort study
Source: Sci Rep. 2022 Aug 1;12:13207. doi: 10.1038/s41598-022-17514-3 (PMC9342835; doi:10.1038/s41598-022-17514-3)
Supplement: Supplementary file 1 — Supplementary Information. [file 41598_2022_17514_MOESM1_ESM.pdf]

Sputnik-V reactogenicity and immunogenicity in the blood and mucosa: a prospective cohort study.

Sergey Yegorov[1,2^], Irina Kadyrova[3^], Baurzhan Negmetzhanov[2,4], Yevgeniya Kolesnikova[3], Svetlana Kolesnichenko[3], Ilya Korshukov[3], Yeldar Baiken[2,4,5], Bakhyt Matkarimov[4], Matthew S. Miller[1], Gonzalo H. Hortelano[2], Dmitriy Babenko[3].

APPENDIX.

This supplemental material has been provided by the authors to give readers additional information about their work.

Table S1. Description of the analytes assessed by multiplex ELISA.

#	Analyte	Functional category	Full name	Alternative name	Lowest limit of detection (pg/ml)
1	IL-1α		Interleukin-1α	-	9.4
2	IL-1β		Interleukin 1β	-	0.8
3	IL-6		Interleukin 6	-	0.9
4	TNF α		Tumor necrosis factor α	DIF	0.7
5	TNF β		Tumor necrosis factor-β	Lymphotoxin-α (LT-α)	1.5
6	IL-17A		Interleukin 17a	CTLA8	0.7
7	sCD40L	Proinflammatory	CD40 ligand	IGM; IMD3; TRAP; CD154;	5.1
8	IL-2		Interleukin 2	TCGF	1.0
9	IL-7	Homeostatic	Interleukin 7	-	1.4
10	IL-12p40		Interleukin-12 p40	Interleukin-23	7.4
11	IL-12P70		Interleukin 12 p70	CLMF; NKSF; CLMF2; IMD28; IMD29; NKSF2;	0.6
12	IL-4		Interleukin 4	BSF1	4.5
13	IL-5		Interleukin 5	EDF; TRF	0.5
14	IL-13	Th1/Th2	Interleukin 13	P600	1.3
15	IL-9	Th9	Interleukin 9	P40; HP40	1.2
16	IL-1Ra		Interleukin-1 receptor antagonist	DIRA; IRAP; IL1F3; IL1RA; MVCD4;	8.3
17	IL-10	Anti-inflammatory	Interleukin 10	CSIF; TGIF; GVHDS	1.1
18	TGF-α		Transforming growth factor α	-	0.8
19	IFN-α2		Interferon α2	-	2,9
20	IFN-γ	Interferons	Interferon γ	IFG; IFI	0.8
21	IL-3		Interleukin 3	MCGF; MULTI-CSF	0.7
22	IL-15		Interleukin 15	-	1.2
23	EGF		Epidermal growth factor	URG; HOMG4	2,8
24	FGF-2		Fibroblast growth factor 2	BFGF; FGFB; HBGF-2	7,6
25	PDGF AA	Growth factors	Platelet-derived growth factor AA	PDGF1	0.4

26	PDGF AB/BB		Platelet-derived growth factor AB/BB	PDGF2	2.2
27	VEGF A		Vascular endothelial growth factor A	VPF; MVCD1	26.3
28	Fit-3L		Fms-related tyrosine kinase 3 ligand	CD135	5.4
29	G-CSF	Colony-stimulating factors	Granulocyte-colony stimulating factor	Colony-stimulating factor 3	1.8
30	GM-CSF		Granulocyte-macrophage colony-stimulating factor	Colony-stimulating factor 2	7.5
31	IL-8	Chemokines	Interleukin 8	CXCL8	0.4
32	Eotaxin		Eosinophil chemotactic protein	CCL11	4,0
33	Fractalkine		Fractalkine	CX3CL1, neurotactin	22.7
34	GRO		human growth-regulated oncogene	CXCL1-3	9.9
35	IP-10		Interferon gamma-induced protein 10	CXCL10	8.6
36	MCP-3		Monocyte-chemotactic protein 3	CCL7	3.8
37	MCP-1		Monocyte-chemotactic protein 1	CCL2	1.9
38	MDC		Macrophage-derived chemokine	CCL22	3.6
39	MIP-1α		Macrophage inflammatory protein 1-α	CCL3	2.9
40	MIP-1β		Macrophage inflammatory protein 1- β	CCL4	3.0
41	RANTES		Regulated on activation, normal t cell expressed and secreted	CCL5	1.2

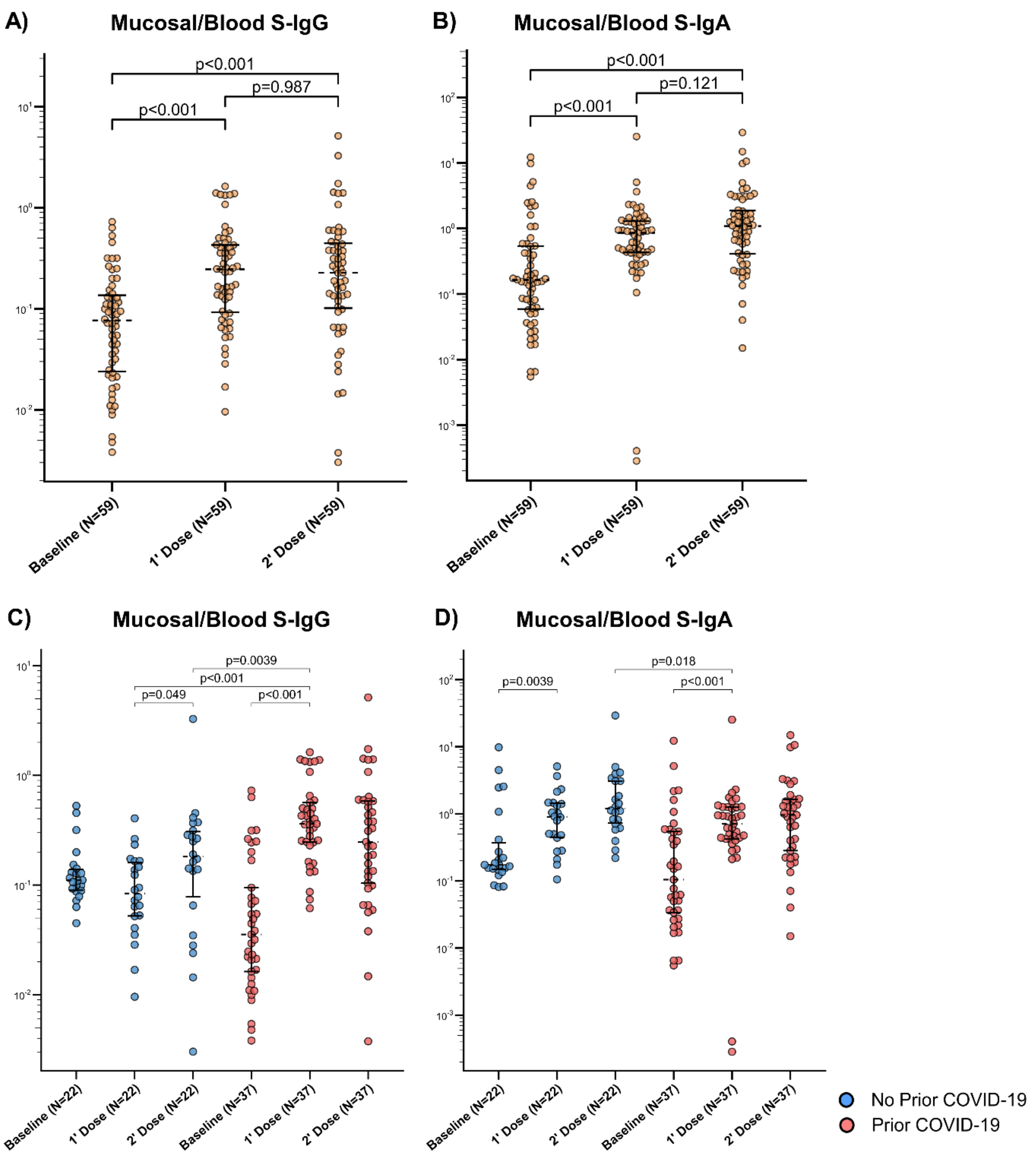

Figure S1. Effects of Sputnik-V vaccination on the ratios of mucosal-to-blood SARS-CoV-2 antibodies in all participants (A,B) and stratified by prior exposure to COVID-19 (C,D). Brackets represent geometric means and 95% confidence intervals; p values indicate the statistical significance assessed by the Mann-Whitney U test. Non-significant p values omitted for secondary analyses in C, D.

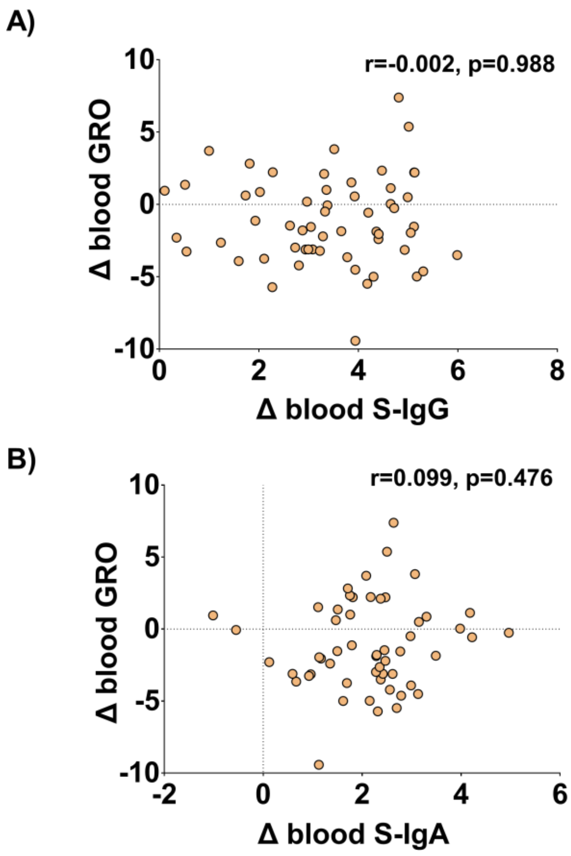

Figure S2. Correlation plots of blood GRO (y-axis) and blood S-IgG (top) and S-IgA (bottom) (x-axis) ratios. Post-Dose 2/baseline ratios were log2-transformed. The Spearman coefficients (r) and their statistical significance (p) are shown.

Table S2. Characteristics of participants with full blood count data available for both baseline and post-dose 2' visits.

**Characteristic**	**Overall, N = 34**	**No prior COVID, N = 9**	**Prior COVID, N = 25**	**p-value***
Age, years, median (IQR)	44.0 (37.8, 53.0)	40.0 (37.0, 52.0)	44.0 (41.0, 53.0)	0.598
Male sex, n (%)	12 (35.3%)	2 (22.2%)	10 (40.0%)	0.439
BMI, kg/m^2, median (IQR)	24.8 (22.9, 25.9)	23.7 (22.5, 28.8)	25.1 (24.0, 25.8)	0.648
Kazakh ethnicity, n (%)	20 (58.8%)	4 (44.4%)	16 (64.0%)	0.435
Any comorbidities	16 (47.1%)	4 (44.4%)	12 (48.0%)	>0.999

* Differences between the Prior and No Prior COVID-19 groups were assessed using Mann-Whitney U or Pearson's Chi-squared tests.

Table S3. List of reactogenicity and immunogenicity studies of Sputnik-V as of 13 January 2022.

**Outcomes**	**Countries**	**References**
**Post-vaccination adverse events**	Argentina, Bosnia and Herzegovina, Iran, Russia, Serbia, San-Marino, Venezuela	[1–7]
**Vaccine-elicited immunologic changes:**		
S/RBD-specific ab titres	Argentina, Bosnia and Herzegovina, Iran, Pakistan, Russia, Serbia, Sri Lanka, Venezuela	[1,2,5,6,8–16]
Other: systemic cytokines	Russia	[11]
Other: cellular assays/immunospot	Russia, Sri Lanka	[1,2,10]

References

1 Logunov DY, Dolzhikova IV, Zubkova OV, *et al.* Safety and immunogenicity of an rAd26 and rAd5 vector-based heterologous prime-boost COVID-19 vaccine in two formulations: two open, non-randomised phase 1/2 studies from Russia. *The Lancet* 2020; **396**: 887–97.

2 Logunov DY, Dolzhikova IV, Shcheblyakov DV, *et al.* Safety and efficacy of an rAd26 and rAd5 vector-based heterologous prime-boost COVID-19 vaccine: an interim analysis of a randomised controlled phase 3 trial in Russia. *The Lancet* 2021; **397**: 671–81.

3 Pagotto V, Ferloni A, Mercedes Soriano M, *et al.* Active monitoring of early safety of Sputnik V vaccine in Buenos Aires, Argentina. *Medicina (B Aires)* 2021; **81**: 408–14.

4 Montalti M, Soldà G, Valerio ZD, *et al.* ROCCA study protocol and interim analysis on safety of Sputnik V vaccine (Gam-COVID-Vac) in the Republic of San Marino: an observational study using active surveillance. 2021.

5 Babamahmoodi F, Saeedi M, Alizadeh-Navaei R, *et al.* Side effects and Immunogenicity following administration of the Sputnik V COVID-19 vaccine in health care workers in Iran. *Sci Rep* 2021; **11**: 21464.

6 Adjobimey T, Meyer J, Sollberg L, *et al.* Comparison of IgA, IgG and neutralizing antibody responses following immunization with Moderna, BioNTech, AstraZeneca, Sputnik-V, Johnson and Johnson, and Sinopharm's COVID-19 vaccines. In Review, 2021 DOI:10.21203/rs.3.rs-1197023/v1.

7 Zare H, Rezapour H, Mahmoodzadeh S, Fereidouni M. Prevalence of COVID-19 vaccines (Sputnik V, AZD-1222, and Covaxin) side effects among healthcare workers in Birjand city, Iran. *International Immunopharmacology* 2021; **101**: 108351.

8 Jeewandara C, Aberathna IS, Danasekara S, *et al.* Comparison of the Immunogenicity of five COVID-19 vaccines in Sri Lanka. 2021.

9 Saeed U, Uppal SR, Piracha ZZ, *et al.* Evaluation of SARS-CoV-2 spike antibody levels among Sputnik V first dose vaccinated people in Pakistan: formulation of national anti-COVID-19 mass vaccination strategy. 2022; published online Jan 15. DOI:10.21203/rs.3.rs-480406/v1.

10 Malavige G, Jeewandara C, Fernando H, *et al.* Immune responses following the first dose of the Sputnik V (Gam-COVID-Vac). 2022; published online Jan 15. DOI:10.21203/rs.3.rs-787293/v2.

11 Martynova E, Hamza S, Garanina EE, *et al.* Long Term Immune Response Produced by the SputnikV Vaccine. *International Journal of Molecular Sciences* 2021; **22**: 11211.

12 Ikegame S, Siddiquey MNA, Hung C-T, *et al.* Neutralizing activity of Sputnik V vaccine sera against SARS-CoV-2 variants. *Nat Commun* 2021; **12**: 4598.

13 Gentile A, Castellano VE, Weinberger N, *et al.* SARS-CoV-2 Antibody Response Following SPUTNIK V Vaccination in Healthcare Workers From a Hospital in Argentina: Preliminary Results. Rochester, NY: Social Science Research Network, 2021 DOI:10.2139/ssrn.3929409.

14 Rossi AH, Ojeda DS, Varese A, *et al.* Sputnik V vaccine elicits seroconversion and neutralizing capacity to SARS-CoV-2 after a single dose. *Cell Rep Med* 2021; **2**: 100359.

15 Chahla RE, Tomas-Grau RH, Cazorla SI, *et al.* Long-term analysis of antibodies elicited by SPUTNIK V: A prospective cohort study in Tucumán, Argentina. *The Lancet Regional Health – Americas* 2022; **6**. DOI:10.1016/j.lana.2021.100123.

16 Claro F, Silva D, Rodriguez M, Rangel HR, Waard JH de. Immunoglobulin G antibody response to the Sputnik V vaccine: previous SARS-CoV-2 seropositive individuals may need just one vaccine dose. *International Journal of Infectious Diseases* 2021; **111**: 261–6.
